# 

**Published:** 1881-01

**Authors:** 


CONTENTS:

| | | Page. |
| --- | --- | --- |
| ***ORIGINAL.*** | | |
| Salutatory, | Editor, | 1 |
| In Memorian, | Ad. Lippe, M. D., Phila. | 3 |
| C. Hering, | Edward Bayard, M.D., New York. | 10 |
| The Organon, Section 153, | P. P. Wells, M.D., Brooklyn, | 15 |
| Homœopathy, | Ad. Fellger, M.D,, Phila. | 22 |
| The Law: Is That All? | C. Pearson, M.D., Washington. | 29 |
| Calendula, | C. Carlton Smith, M.D., Phila. | 31 |
| Honor to Whom Honor Is Due, | | 35 |
| ***BOOK NOTICES AND REVIEWS.*** | | |
| Diphtheria, By Rollen R. Gregg, M.D., Buffalo. | | 37 |
| Report of Am. Ins. of Homœopathy, | | 37 |

